# Theoretical study of the adsorption of benzene on coinage metals

**DOI:** 10.3762/bjoc.10.185

**Published:** 2014-08-04

**Authors:** Werner Reckien, Melanie Eggers, Thomas Bredow

**Affiliations:** 1 Mulliken Center for Theoretical Chemistry, Rheinische Friedrich-Wilhelms-Universität Bonn, Beringstr. 4, 53115 Bonn, Germany

**Keywords:** adsorption, benzene, coinage metals, density functional theory, dispersion correction, template

## Abstract

The adsorption of benzene on the M(111), M(100) and M(110) surfaces of the coinage metals copper (M = Cu), silver (M = Ag) and gold (M = Au) is studied on the basis of density functional theory (DFT) calculations with an empirical dispersion correction (D3). Variants of the Perdew–Burke–Ernzerhof functionals (PBE, RPBE and RevPBE) in combination with different versions of the dispersion correction (D3 and D3(BJ)) are compared. PBE-D3, PBE-D3(BJ) and RPBE-D3 give similar results which exhibit a good agreement with experimental data. RevPBE-D3 and RevPBE-D3(BJ) tend to overestimate adsorption energies. The inclusion of three-center terms (PBE-D3(ABC)) leads to a slightly better agreement with the experiment in most cases. Vertical adsorbate–substrate distances are calculated and compared to previous theoretical results. The observed trends for the surfaces and metals are consistent with the calculated adsorption energies.

## Introduction

The adsorption of organic molecules on metals is of great interest since the formation of thin films and self-assembled monolayers opens the way toward a functionalization of surfaces [1–8]. The adsorbed molecules often contain an aromatic framework that can be substituted with functional groups. The bonding between the surface and the adsorbate is an interplay between electrostatic interaction, including charge transfer (CT) to the surface, and covalent contributions [9–11]. In addition, it was found that dispersion interaction plays a crucial role for the adsorption of large aromatic compounds on metal surfaces [9–11]. This holds in particular for the adsorption on the coinage metals copper, silver and gold. Therefore, a theoretical treatment of this process requires methods that provide an accurate description of these weak interactions. Density functional theory (DFT) is established as a standard method for quantum-chemical solid-state calculations [12]. However, DFT has the well known shortcoming that it fails to describe dispersion effects. Consequently, standard DFT methods are not suitable for the calculation of the adsorption of aromatic compounds. In the last years much effort has been directed to the development of DFT methods that eliminate this shortage [13–24]. One of them is a damped empirical correction called DFT-D3 which was proposed by Grimme et al. for molecular systems [13]. The D3-dispersion correction to the DFT energy is calculated by summation over pair potentials. Non-additive effects of dispersion interaction can be treated on the basis of three-body terms D3(ABC) [13]. The most recent DFT-D3(BJ) method [16] differs from the original DFT-D3 essentially only in the damping function for short range interaction. Due to the computational efficiency of the D3 correction schemes it is possible to perform DFT-D3 calculations with nearly the same computational effort as standard DFT calculations. Only the calculation of three-body terms can become expensive for large systems, and is therefore usually carried out only for the final energy estimation. For molecular systems one can obtain with DFT-D3 results that are close to coupled cluster singles doubles with perturbative triples (CCSD(T)) results at the cost of GGA-DFT calculations. In recent years the DFT-D3 method has been extended to periodic systems [25]. In a few cases [26–27] it is observed that the metal *C*_6_ dispersion coefficients for bulk systems can be largely reduced compared to the values of free atoms. Indeed this does not apply to the coinage metals Cu, Ag, and Au. The *C*_6_ parameters for these metals are already converged [28]. In addition, it is known that metal substrates show significant dispersion screening effects that can modify the polarizabilities and *C*_6_ coefficients of adsorbed molecules [29–30]. In principle these effects should be included in the coordination number dependent *C*_6_ coefficients of the D3 correction. We checked this by calculating the *C*_3_ coefficients for the benzene adsorption on the Au(111) surface.

Previous theoretical studies of the adsorption of organic compounds on silver and gold surfaces resulted in a good agreement with experimental results [9–11,26]. However, a systematic comparison of the different DFT-D3 approaches is still missing. One aim of this work is the comparison of different DFT-D3 methods for the description of the adsorption of aromatic compounds on this surfaces. We therefore present a theoretical study of the adsorption of benzene on the M(111), M(100) and M(110) surfaces of the coinage metals copper, silver and gold. The benzene molecule was selected because it is a building block of many organic compounds that are used for surface functionalization. As mentioned above the binding between the metal and the adsorbate is often dominated by dispersion interaction to the aromatic framework. Therefore the study of the bonding between benzene and the metal surfaces is of great interest. In addition, although the adsorption of benzene on some of these surfaces has been subject of previous theoretical studies [29–39], this is the first work in which the adsorption on the most important coinage metal surfaces is systematically studied with the same method. Different from our previous study on benzene/Ag(111) [25], we apply a variety of DFT methods and dispersion corrections, and investigate all low-index surfaces.

## Computational methodologies

We used the plane-wave code VASP [40–42] in combination with the projector-augmented wave method to account for the core electrons [43] for all calculations. We applied our recent implementation [25] of Grimme’s dispersion correction (DFT-D3) [13,16]. The dispersion corrected DFT-D3 energy *E*_DFT−D_ is calculated by adding an empirical correction energy *E*_disp_ to the DFT energy *E*_DFT_, see Equation 1.

[1]
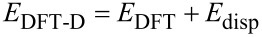


In this work we used the gradient-corrected PBE [44], RPBE [45], and RevPBE [46] functionals in combination with the original D3 [13] as well as with the newer D3(BJ) dispersion correction [16]. These methods were chosen since they represent a selection of standard GGA functionals, which are available in most of the software for quantum chemical solid state studies. The computational effort of hybrid functionals is too large for these systems and cheaper methods like DFTB-D3 [47] lack suitable parameters, e.g., for gold. We also checked the impact of the three-body terms (D3(ABC)). These three-center terms D3(ABC) were introduced in the D3 correction scheme since the long-range part of the interaction between three ground-state atoms is not exactly equal to the pairwise interaction energies [13]. From third-order perturbation theory one gets the Axilrod–Teller–Muto dispersion term for the consideration of the non-additivity of dispersion interaction. D3(ABC) is calculated according to Equation 2

[2]
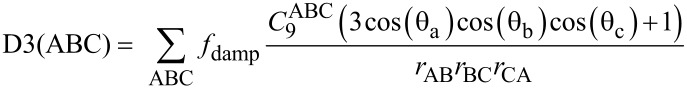


The 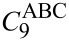 coefficients are the geometric mean of the *C*_6_-coefficients, θ_a_, θ_b_ and θ_c_ are the angles of the triangle, which is formed by the three atoms, and *f*_damp_ is the damping function. The Axilrod–Teller–Muto dispersion terms can be neglected for molecular systems [13]. However, recent studies indicate that their impact is larger for periodic systems that are more densely packed.

We performed calculations with the PBE-D3, PBE-D3(BJ), PBE-D3(ABC), RPBE-D3, RevPBE-D3, and RevPBE-D3(BJ) approaches. We used a cutoff energy of 400 eV for the plane-wave valence basis and a 3 *×* 3 *×* 1 *k*-point mesh for reciprocal-space integration. Preliminary convergence studies showed that these values are an optimal compromise between accuracy and computational efficiency. The calculated structure parameters and adsorption energies changed by several mÅ and kJ/mol, respectively, when larger cutoff energies 600 eV and 900 eV were used. However, the reported trends did not change.

The systems investigated are formed by the clean, unreconstructed M(100), M(111), or M(110) surfaces as substrate and one benzene molecule as adsorbate. We chose a


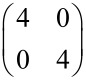


supercell with four atomic layers for the adsorption on the M(100) and M(111) surfaces and a


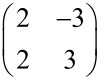


supercell with seven atomic layers for the adsorption on the M(110) surfaces. For each surface we performed calculations for different adsorption sites and orientations of the benzene molecule.

Geometry optimizations were performed with the PBE-D3 method, which was already used for the study of PTCDA on the Ag(111), Ag(100) and Ag(110) surfaces [9]. For these calculations we chose an energy convergence criterion of 10^−6^ eV for the SCF energy, and of 5*·*10^−3^ eV/Å for the ionic relaxation (forces are converged if smaller than 5*·*10^−3^ eV/Å). The first three atomic layers of M(100) and M(111) and the first five atomic layers of M(110) were relaxed while the atoms of the lowermost layers were kept at their bulk-like positions. We give two different values for the adsorption distance. *d*_1_ is the vertical distance between the adsorbate and the topmost layer of the surface. The adsorption of benzene effects a slight relaxation of the surface. Therefore we calculate *d*_1_ with respect to the mean value of the *z*-coordinates. *d*_2_ is the distance between the adsorbate and the hypothetical topmost surface layer for an unrelaxed surface. The latter is given since this enables a comparison to data which have been derived from normal incidence X-ray standing waves (NIX-SW) spectroscopy. This may be useful for a comparison with future experimental results, similar to our previous studies [9–11]. Both distances are determined by calculating the difference between the averaged *z*-coordinate of the carbon atoms and the surface atoms.

Potential curves are obtained on the basis of single-point calculations with the PBE-D3, PBE-D3(BJ), PBE-D3(ABC), RPBE-D3, RevPBE-D3(BJ) and RPBE-D3 approaches. We stepwise altered the distance between the benzene molecule and the unrelaxed surface from 2.5 Å to 5.0 Å. Since *d*_1_ and *d*_2_ do not differ for the potential curve we give only one adsorption distance *d*. Adsorption energies *E*_ads_ are calculated according to the supramolecular approach: *E*_ads_ = *E*(system) − *E*(surface) − *E*(adsorbate). A Bader analysis [48–49] was performed in order to study the net charge transfer between the surfaces and the adsorbed molecule.

## Results and Discussion

First we identified the most stable adsorption sites on the basis of PBE-D3 optimizations by placing the benzene molecule on different adsorption sites and calculating the adsorption energies. The following discussion is limited to the most stable adsorption sites for each surface. Other adsorption sites are higher in energy by 2 to 15 kJ/mol. This points to a relatively high mobility of benzene on the surfaces assuming that the corresponding activation barriers are of similar magnitude. We did not calculate the transition states because this was not the scope of the present study. We also checked that the orientation of the flat lying molecules with respect to the underlying surfaces has no significant influence to the adsorption energies and geometries: A stepwise rotation of the molecule around the *z*-axis changes *E*_ads_ by less than 1 kJ/mol in each case.

**Figure 1:**
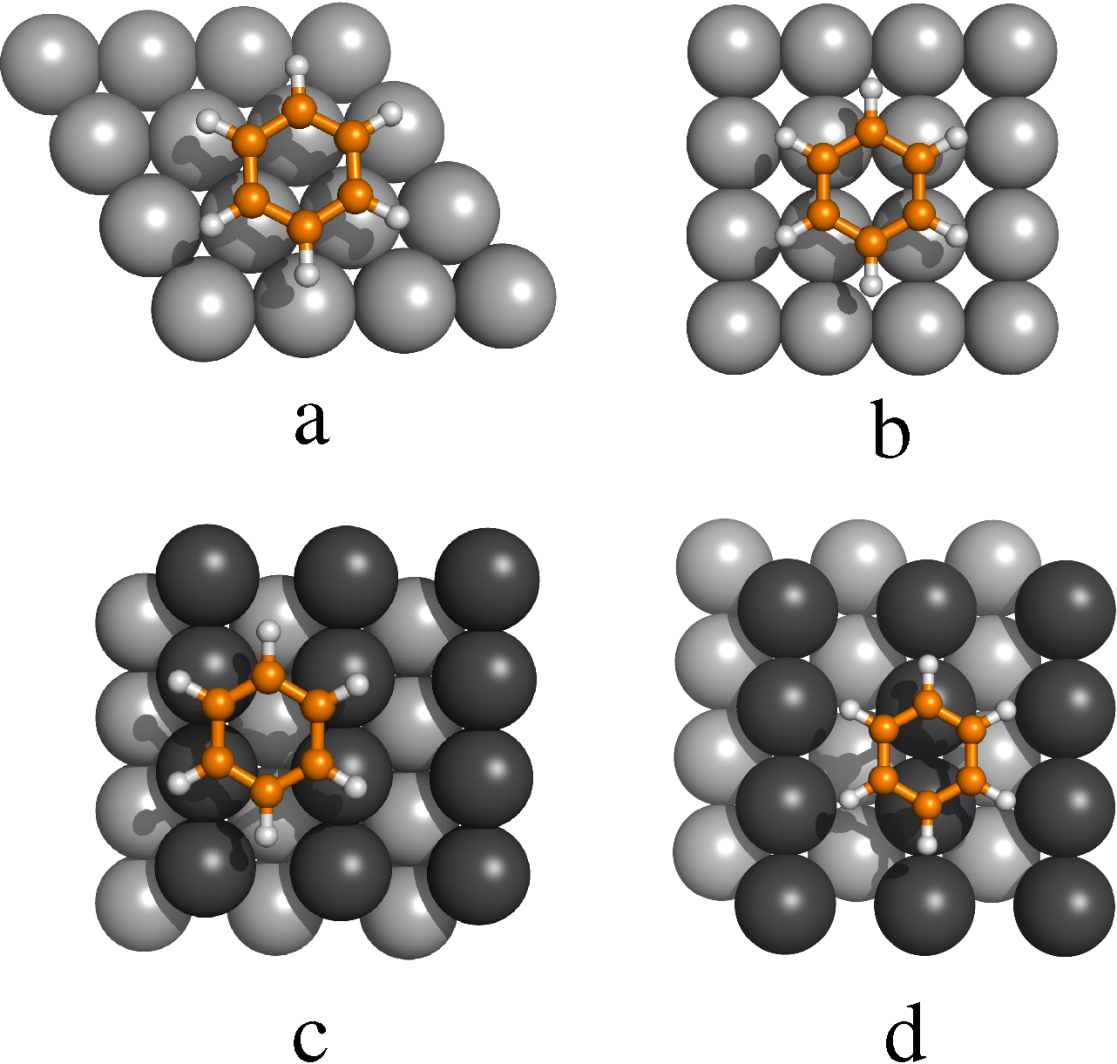
Preferred adsorption sites for benzene: threefold hollow for M(111) (a), fourfold hollow for M(100) (b), fourfold hollow for Cu(110) (c), and bridge for M(110) (d).

The results of the optimizations are summarized in Table 1. It was found that all metals have the same preferred adsorption sites for the M(111) and M(100) surfaces, the threefold hollow site for M(111) and the fourfold hollow site for M(100), see Figure 1. As a general trend we observe that structures with shorter C–metal distances are more stable than the others. For the M(110) surface it was found that Cu prefers benzene adsorption on a fourfold hollow position, whereas Ag and Au prefer a bridge position, see Figure 1. The notation of the adsorption places refers to the center of the benzene molecule.

**Table 1:** Adsorption energies *E*_ads_ in kJ/mol and adsorption distances *d*_1_ and *d*_2_ in Å obtained with the PBE-D3 functional. *d*_1_ is calculated with respect to the topmost layer of the surface, *d*_2_ (estimated with respect to the unrelaxed surface) is for the sake of comparison to NIX-SW experiments.

surface	*E*_ads_			*d*_1_			*d*_2_		

	Cu	Ag	Au	Cu	Ag	Au	Cu	Ag	Au

(111)	−97	−72	−85	2.86	3.17	3.10	2.87	3.19	3.18
(100)	−114	−75	−87	2.45	3.00	2.93	2.47	3.04	3.01
(110)	−117	−76	−85	2.35	2.78	2.84	2.33	2.80	2.84

**Figure 2:**
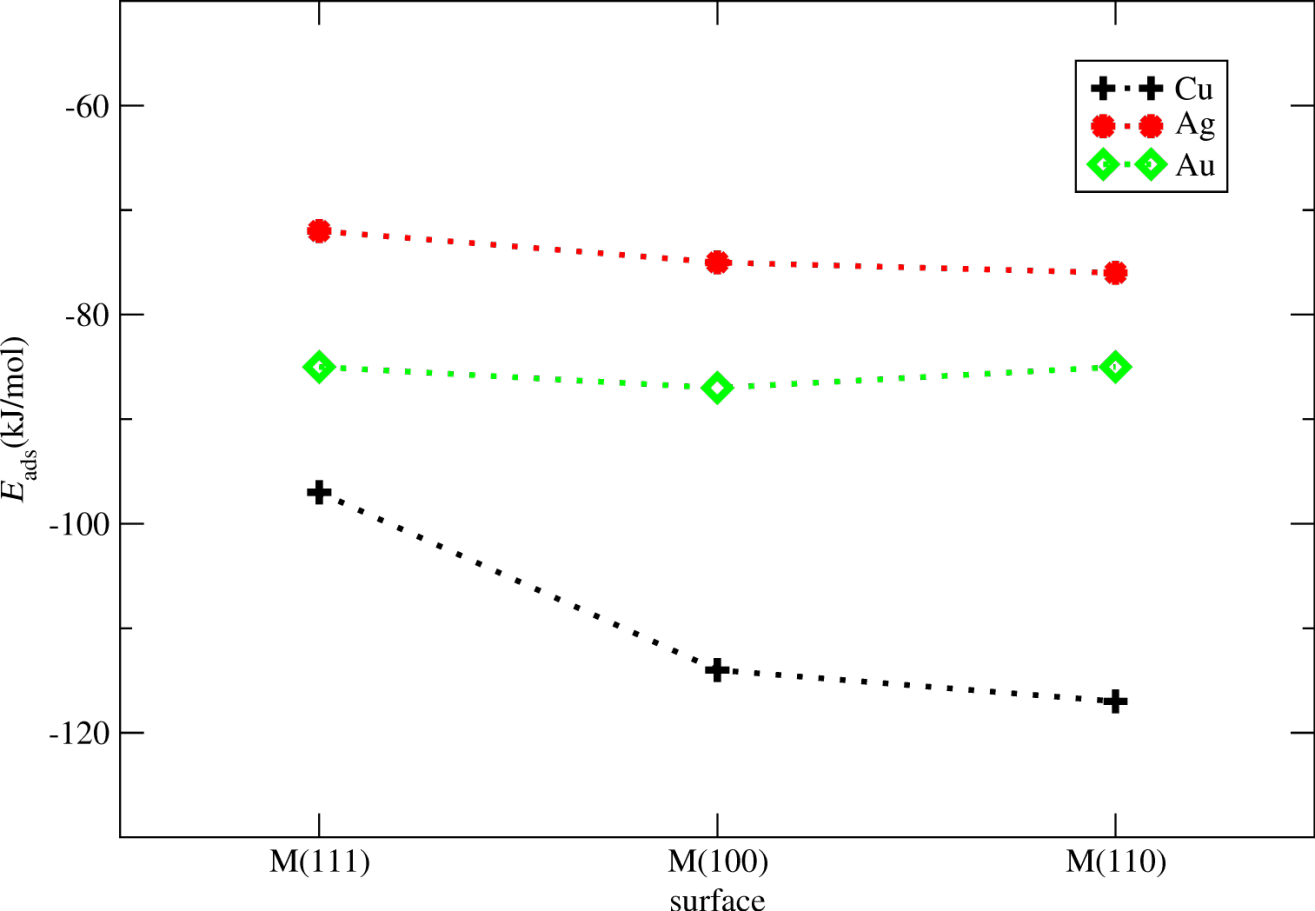
Benzene on Cu, Ag, and Au surfaces. Calculated adsorption energies in kJ/mol for M(111), M(100), and M(110), M = Cu, Ag, Au.

The binding between benzene and copper is the largest of all investigated surfaces. *E*_ads_ is calculated to *E*_ads_ = −117 kJ/mol for Cu(110), *E*_ads_ = −114 kJ/mol for Cu(100) and *E*_ads_ = −97 kJ/mol for Cu(111). For gold the adsorption energies are *E*_ads_ = −85 kJ/mol for the Au(110), *E*_ads_ = −87 kJ/mol for the Au(100), and *E*_ads_ = −85 for the Au(111) surface. The lowest adsorption energies are calculated for the silver surfaces, *E*_ads_ = −76 kJ/mol for Ag(110), *E*_ads_ = −75 kJ/mol for Ag(100) and *E*_ads_ = −72 kJ/mol for Ag(111). It is worth to mention that the adsorption energies on a given metal are almost independent from the surface type. The sole exception is the Cu(110) surface the adsorption energy of which is about −20 kJ/mol smaller than *E*_ads_ on Cu(100) and Cu(110). For all other systems we find that the adsorption energies on the selected surface planes are within the range of 3 kJ/mol. The results are summarized in Figure 2.

In contrast we observe larger variations for the adsorption distances *d*_1_ and *d*_2_. We limit the discussion to the *d*_1_ values. The *d*_2_ values are only given in order to enable a comparison to future NIX-SW studies. We observe that *d*_1_ is smallest for the (110) surfaces and largest for the (111) surfaces. By comparison of the metals we observe, that the adsorption distances are shortest for the copper surfaces and longest for the silver surfaces. The adsorption distances are *d*_1_ = 2.86 Å for Cu(111), *d*_1_ = 2.45 Å for Cu(100), *d*_1_ = 2.35 Å for Cu(110), *d*_1_ = 3.17 Å for Ag(111), *d*_1_ = 3.00 Å for Ag(100), *d*_1_ = 2.78 Å for Ag(110), *d*_1_ = 3.10 Å for Au(111), *d*_1_ = 2.93 Å for Au(100) and *d*_1_ = 2.84 Å for Au(110). This trend is consistent with the calculated adsorption energies, which are smallest for silver and largest for copper. Unfortunately, to the best of our knowledge no experimentally determined distances are available for these systems. However, previous experimental and theoretical studies of PTCDA on the Ag(111), Ag(100) and Ag(110) surfaces [9–10] indicate that the PBE-D3 and PBE-D3(BJ) approaches give accurate adsorption distances.

**Figure 3:**
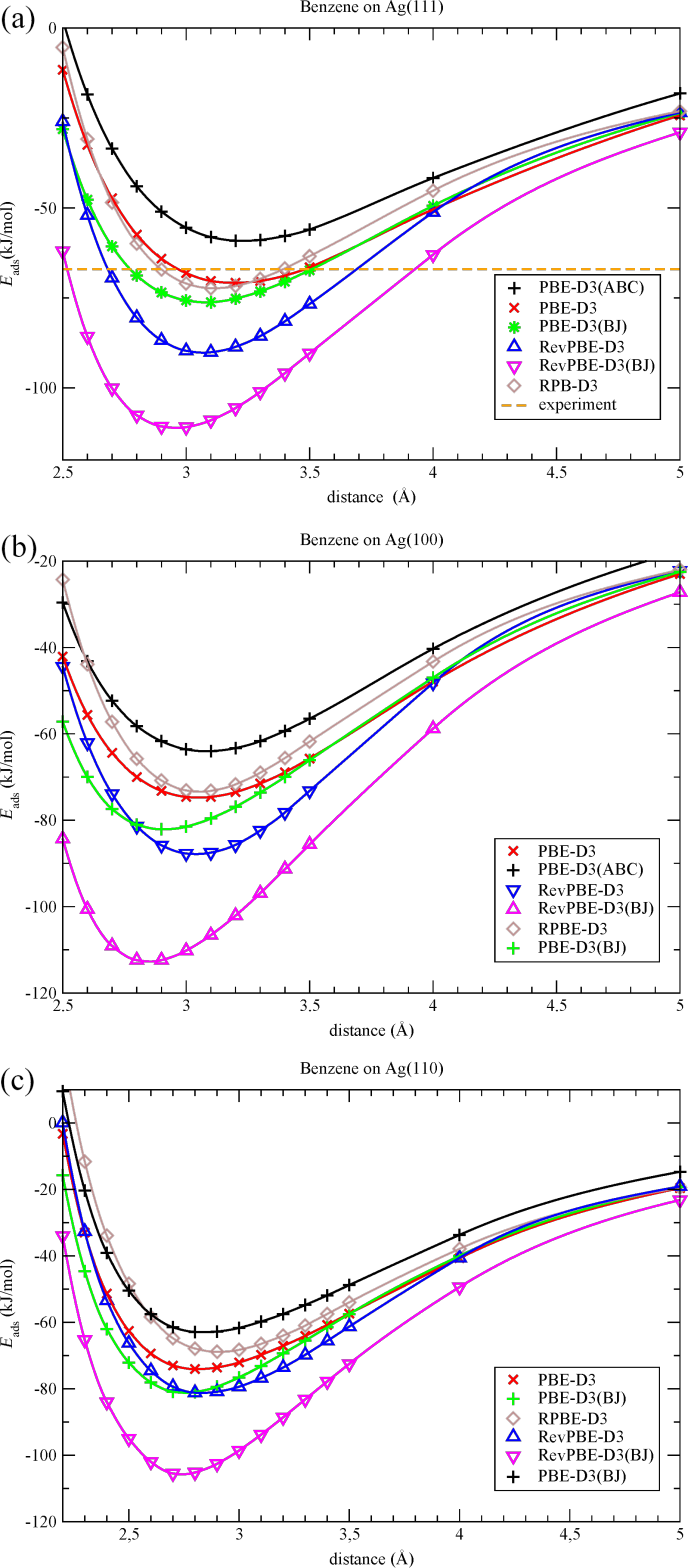
Potential curves for the adsorption of benzene on the (a) Ag(111), (b) Ag(100), and (c) Ag(110) surfaces.

A closer look at the optimized structures reveals that the benzene molecule and the underlying surfaces are only slightly affected by adsorption. Therefore, the following comparison of different DFT-D approaches has been performed on the basis of potential curves with fixed structures of benzene and surface. The results of these calculations are summarized in Table 2. The potential curves for the silver surfaces are shown in Figure 3. The potential curves for the copper and gold surfaces are included in Supporting Information File 1.

**Table 2:** Results for the adsorption of benzene obtained from potential curves. *E*_ads_ is given in kJ/mol, *d* is given in Å.

	Cu(111)		Cu(100)		Cu(110)	
	*E*_ads_	*d*	*E*_ads_	*d*	*E*_ads_	*d*

PBE-D3	−96	2.88	−110	2.58	−111	2.46
PBE-D3(BJ)	−93	2.85	−108	2.58	−113	2.44
RPBE-D3	−100	2.81	−108	2.60	−104	2.54
RevPBE-D3	−127	2.73	−139	2.52	−126	2.50
RevPBE-(BJ)	−143	2.71	−154	2.56	−150	2.44
PBE-D3(ABC)	−76	2.95	−99	2.61	−93	2.49
exp.	−69	—		—	−99	—

	Ag(111)		Ag(100)		Ag(110)	
	*E*_ads_	*d*	*E*_ads_	*d*	*E*_ads_	*d*

PBE-D3	−71	3.20	−75	3.05	−74	2.82
PBE-D3(BJ)	−76	3.08	−82	2.91	−81	2.76
RPBE-D3	−72	3.12	−73	3.06	−69	2.91
RevPBE-D3	−91	3.07	−88	3.04	−81	2.84
RevPBE-(BJ)	−111	2.96	−112	2.85	−105	2.74
PBE-D3(ABC)	−59	3.24	−64	3.09	−63	2.84
exp.	−67	—		—		—

	Au(111)		Au(100)		Au(110)	
	*E*_ads_	*d*	*E*_ads_	*d*	*E*_ads_	*d*

PBE-D3	−83	3.16	−87	3.04	−92	2.72
PBE-D3(BJ)	−84	3.08	−89	2.94	−95	2.69
RPBE-D3	−84	3.10	−85	3.03	−84	2.81
RevPBE-D3	−105	3.07	−103	3.01	−101	2.73
RevPBE-(BJ)	−121	2.98	−123	2.87	−123	2.69
PBE-D3(ABC)	−70	3.19	−74	3.06	−79	2.73
exp.	−73	—		—		—

In all cases the vertical distances obtained with the potential curves for PBE-D3 are in good agreement with the results of the full geometry optimization. This confirms the validity of the simplified approach.

It was found, that PBE-D3 and PBE-D3(BJ) give similar results for all surfaces. The PBE-D3(BJ) adsorption energies tend to be about 4 kJ/mol larger in absolute value than the PBE-D3 energies. This behavior is to be expected: Both methods essentially only differ in the empirical damping function for short-range interaction. The RPBE-D3 curves are quite similar to the PBE-D3 curves. Some deviations are found only for the M(110) surfaces. The difference is less than 8 kJ/mol for all systems. RevPBE and RevPBE-D3(BJ) show larger deviations from the PBE-D3 results. The RevPBE-D3(BJ) adsorption energies are in the range of −31 to −47 kJ/mol, *E*_ads_(RevPBE-D3) is 7 to 31 kJ/mol more negative than *E*_ads_(PBE-D3). Both approaches overestimate the interaction between benzene and the metal surfaces, in particular RevPBE-D3(BJ). In the second to last line of Table 2 we give the results of PBE-D3(ABC) calculations. The three-body correction to dispersion is repulsive in this case. This is in line with a previous study of the influence of the three-body terms to periodic systems. The PBE-D3(ABC) adsorption energies are 11 to 20 kJ/mol less negative than the PBE-D3 energies. As expected it was found that none of the pure DFT functionals is able to give a correct description of the adsorption. Most potential curves (not shown) are repulsive over the entire distance range. Only the potential curves obtained with the PBE functional exhibit some very flat minima in the range of −10 kJ/mol at larger distances. Accordingly the calculated adsorption energies can be almost solely ascribed to the dispersion correction. The contribution of *E*_disp_ to the adsorption energy is larger than 90% for all systems. This confirms that the surface–adsorbate interaction is dominated by dispersion interaction.

Nevertheless we also calculated the charge transfer between benzene and the metal surfaces on the basis of a Bader analysis in order to investigate electrostatic contributions to the interactions. For the Cu(111), Ag(111) and all gold surfaces we observe only a small charge transfer, between 0.02 a.u. (for Ag(111)) and 0.12 a.u. (for Au(100)) from benzene to the metal surface. For the other systems we calculate a small charge transfer from the surface to the benzene molecule in the range of 0.03 (for Ag(100)) to 0.07 a.u. (for Cu(110)). As expected this charge transfer is much smaller than the one between functionalized aromatic compounds and coinage metal surfaces [9,11]. The different direction of the charge transfer may be explained by the work function of the metal surfaces. Systems which exhibit a charge transfer to the metal have a larger work function as the systems, in which the charge transfer takes is directed to the adsorbate. The calculated work functions are 4.07 eV for Ag(100), 4.08 eV for Cu(110), 4.13 eV for Ag(110), 4.19 eV for Cu(100), 4.30 eV for Ag(111) 4.49 eV for Cu(111), 4.77 eV for Au(110), 4.88 eV for Au(100) and 4.91 eV for Au(111).

In Table 3 we compare our calculated results obtained with PBE-D3 and PBE-D3(ABC) to available theoretical and experimental data. The theoretical values show a quite large fluctuation range. In a few cases the deviations can be explained by well known shortcomings of the used methods, which are discussed in literature. However, even the recently developed dispersion DFT methods (vdW-DF and PBE+vdW) exhibit fluctuations of up to 30 kJ/mol for adsorption energies and 0.4 Å for adsorption distances. In general, our results are in good agreement with those other theoretical works that include dispersion effects. Experimental adsorption energies are −69 kJ/mol for Cu(111), −67 kJ/mol for Ag(111), −73 kJ/mol for Au(111), and −99 kJ/mol for Cu(110) [50]. PBE-D3 and RPBE-D3 give the best agreement for the Ag(111) surface. These methods overestimate *E*_ads_ by 4 or rather 5 kJ/mol. The deviation of PBE-D3(BJ) (overestimation) and PBE-D3(ABC) (underestimation) are of the order of 10 kJ/mol. It appears that all standard DFT-D methods tend to overestimate *E*_ads_ on the Cu(111) and Au(111) surfaces by at least 10 (Au(111)) to 24 kJ/mol (Cu(111)). The deviations are substantially reduced to −3 kJ/mol for Au(111) and 7 kJ/mol for Cu(111) if the PBE-D3(ABC) method is used. For the Cu(110) surface RPBE-D3 shows the smallest deviation, −5 kJ/mol. PBE-D3 and PBE-D3(BJ) overestimate *E*_ads_ by 12 to 14 kJ/mol, whereas PBE-D3(ABC) leads to a slight underestimation of 6 kJ/mol. Contributions from the zero-point energy and thermal corrections that lower the adsorption energies are not considered in this work. If these are taken into account we conclude that PBE-D3, PBE-D3(BJ) and RPBE-D3 give a good agreement to experimental adsorption energies. For the M(111) surfaces we get a slightly better agreement if the three-center terms are considered. RevPBE-D3 and RevPBE-D3(BJ) differ by up to −58 kJ/mol (RevPBE-D3) and −74 kJ/mol from the experimental results. Therefore we conclude that RevPBE-D3 and RevPBE-D3(BJ) methods are not suited for the calculation of aromatic organic compounds on these metal surfaces. However, the application of the other DFT-D methods treated in this work can be recommended.

**Table 3:** Comparison of adsorption energies *E*_ads_ in kJ/mol and adsorption distances *d* in Å with available theoretical and experimental data.

system	*E*_ads_	*d*	method, source

Cu(111)	−79	2.83	optB86b, [37]
	−71	2.91	optB88, [37]
	−66	3.14	optPBE, [37]
	−51	3.46	revPBE, [37]
	−47	3.39	rPW86, [37]
	−34	3.6	MP2, [35]
	−3	—	PW91, [36]
	−98	3.04	PBE+vdW [29]
	−76	2.79	PBE+vdW^surf^ [29]
	−48	4.14	vdW-DF [29]
	−45	3.38	vdW-DF2 [29]
	−61	3.08	optPBE-vdW [29]
	−66	3.12	optB88-vdW [29]
	−69		opt-B86b-vdW [29]
	−96	2.88	PBE-D3, this work
	−76	2.95	PBE-D3(ABC), this work
	−69	—	experiment, [51–52]

Cu(110)	−109	2.003	VWN, [39]
	−39	—	GGA-DFT [38]
	−111	2.46	PBE-D3, this work
	−93	2.49	PBE-D3(ABC), this work
	−99	—	experiment, [51]

Ag(111)	−72	—	PBE+vdW^surf^, [31]
	−70	—	optB88-vdW, [31]
	−73	3.02	optB86b, [37]
	−70	3.08	optB88, [37]
	−69	3.23	optPBE, [37]
	−53	3.51	revPBE, [37]
	−50	3.40	rPW86, [37]
	−32	3.7	MP2, [35]
	−5	—	PW91, [36]
	−80	3.14	PBE+vdW [29]
	−70	2.96	PBE+vdW^surf^ [29]
	−50	3.95	vdW-DF [29]
	−45	3.40	vdW-DF2 [29]
	−65	3.29	optPBE-vdW [29]
	−69	3.12	optB88-vdW [29]
	−73	3.10	opt-B86b-vdW [29]
	−71	3.20	PBE-D3, this work
	−59	3.24	PBE-D3(ABC), this work
	−67	—	experiment, [52–53]

Au(111)	−83	3.03	optB86b, [37]
	−79	3.08	optB88, [37]
	−69	3.21	optPBE, [37]
	−54	3.44	revPBE, [37]
	−53	3.31	rPW86, [37]
	−71	3.05	PBE+vdW^surf^, [31]
	−76	3.23	optB88-vdW, [31]
	−57	3.44	vdW-DF, [31]
	−54	3.29	vdW-DF2, [31]
	−41	3.7	RPBE-vdW, [32]
	−30	3.8	MP2, [35]
	−8	—	PW91, [36]
	−77	3.21	PBE+vdW [29]
	−70	3.05	PBE+vdW^surf^ [29]
	−57	3.44	vdW-DF [29]
	−54	3.29	vdW-DF2 [29]
	−72	3.22	optPBE-vdW [29]
	−76	3.23	optB88-vdW [29]
	−81	3.12	opt-B86b-vdW [29]
	−83	3.16	PBE-D3, this work
	−70	3.19	PBE-D3(ABC), this work
	−73	—	experiment, [52,54]

Au(100)	−185	2.376	VWN, [34]
	−87	3.04	PBE-D3, this work
	−74	3.06	PBE-D3(ABC), this work

The differences in adsorption distances for the recommended methods are in the range from 0.03 to 0.18 Å. As expected, PBE-D3(ABC) gives the largest distances for all systems due to the repulsive nature of the three-center terms. However, the deviation from the PBE-D3 distance is less than 0.07 Å. Therefore it is possible to neglect these contributions in structure optimizations without significant loss of accuracy, which is advantageous since the calculation of the three-center terms is rather expensive for large systems.

In Table 4 we give the *C*_3_ coefficients for the benzene adsorption on the Au(111) surface. The data are compared to the *C*_3_ coefficient for the PBE+vdW^surf^ functional, which is constructed to reproduce the exact values [29]. The *C*_3_ coefficients are fitted from the pure dispersion interaction term at large distances according to the method described in [29].

**Table 4:** *C*_3_ coefficients in eV·Å^3^ for benzene on the Au(111) surface.

method	*C*_3_	

PBE-D3	12.23 *±* 0.24	
PBE-D3(BJ)	12.23 *±* 0.24	
RPBE-D3	11.95 *±* 0.22	
RevPBE-D3	12.48 *±* 0.27	
RevPBE-D3(BJ)	13.42 *±* 0.49	
PBE-D3(ABC)	3.89 *±* 0.39	
PBE+vdW^surf^	9.16 *±* 0.08	[29]

It was found that PBE-D3, PBE-D3(BJ) RPBE-D3, RevPBE-D3 and RevPBE-D3(BJ) overestimate (by 30%) the *C*_3_ coefficients compared to PBE+vdW^surf^ whereas PBE-D3(ABC) underestimates them (by 50%). It has to be mentioned, however, that the values of the *C*_3_ coefficients strongly depend on the functional. For example, in [29] values between 4 and 9 eV·Å^3^ have been reported. Accordingly, we think that our deviations are within a reliable range.

## Conclusion

The adsorption of benzene on the M(111), M(100) and M(110) surface of the coinage metals copper, silver and gold is studied with different DFT-D3 methods. RevPBE-D3 and RevPBE-D3(BJ) overestimate the surface–adsorbate interaction, PBE-D3, PBE-D3(BJ) and RPBE-D3 give similar results for adsorption energies with better agreement to experimental data. The calculated adsorption energies decrease in the ordering Cu *>* Au *>* Ag and M(110) *>* M(100) *>* M(111). The latter trend can be explained with the increasing coordination number of the surface metal atoms. The higher reactivity of gold compared to silver is attributed to the larger polarizability of the gold atoms. The adsorption distances are almost the same on these two surfaces due to the similar van der Waals radii of 172 pm for Ag [55] and 166 pm for Au [55]. Hence, the larger *C*_6_ coefficients of 317.2 a.u. for the gold atoms [56] (compared to 268.6 a.u. for the silver atoms [56]) result in a larger dispersion interaction between the surface and the substrate. Copper has the smallest polarizability of the three metals. The *C*_6_ coefficients for the surface atoms is 175.0 a.u. within the D3-correction [56]. However, the benzene molecules come closer to the copper surfaces due to the smaller van der Waals radius of 140 pm [55]. From there the dispersion interaction to the copper surface is largest although Cu has the smallest *C*_6_ coefficients. PBE-D3, PBE-D3(BJ) and RPBE-D3 tend to slightly overestimate the adsorption energies in comparison to experiment, in particular for the Cu(111) surface. This effect is reduced when the three-body correction to dispersion is considered. The PBE-D3(ABC) adsorption energies are smaller in absolute value by 10 to 20 kJ/mol compared to the standard PBE-D3 values. This leads in most cases to a slightly better agreement with the available experimental results. As a result of this work we recommend DFT-D methods like PBE-D3, PBE-D3(BJ) or RPBE-D3 for the theoretical study of the adsorption of aromatic compounds on metal surfaces. Due to the high computational cost of the evaluation of three-center terms, we suggest to perform geometry optimizations with PBE-D3 followed by single-point calculations with PBE-D3(ABC) for adsorption energies. Surprisingly, we realize that the RevPBE-D3 and RevPBE-D3(BJ) methods, which yield a more realistic description of the adsorption of small molecules on ionic surfaces [26], seem to be not suitable for the present systems. Since the pure RevPBE potential curves are repulsive one can ascribe the observed overestimation of the adsorption energies solely to the dispersion correction.

We give adsorption distances with respect to the topmost layer of the relaxed surface, and with respect to the hypothetical topmost surface layer for an unrelaxed surface. The latter allows for a comparison to data from NIX-SW spectroscopy. It was found, that vertical distances are smallest for the M(110) surfaces and largest for the M(111) surfaces, in accordance with the trends of the adsorption energies. The distances on the copper surfaces are in the range from 2.35 to 2.86 Å, the distances on the silver surfaces in the range from 2.78 to 3.17 Å, and the distances on the gold surfaces are in the range from 2.84 to 3.18 Å. It will be interesting to compare these data with future experimental data.

## Supporting Information

Supporting information features potential curves for the adsorption on the Cu(111), Cu(100), Cu(110), Au(111), Au(100), and Au(110) surfaces.

File 1 Potential curves for adsorption of benzene on copper and gold surfaces.
