# Supplementary material for: Theoretical study of the adsorption of benzene on coinage metals
Source: Beilstein J Org Chem. 2014 Aug 4;10:1775–84. doi: 10.3762/bjoc.10.185 (PMC4142854; doi:10.3762/bjoc.10.185)
Supplement: File 1 — Potential curves for adsorption of benzene on copper and gold surfaces. [file Beilstein_J_Org_Chem-10-1775-s001.pdf]

Supporting Information

for

Theoretical study of the adsorption of benzene on coinage metals

Werner Reckien, Melanie Eggers and Thomas Bredow*

Address: Mulliken Center for Theoretical Chemistry, Rheinische Friedrich-Wilhelms-Universität Bonn, Beringstr. 4, 53115 Bonn, Germany

Email: Thomas Bredow - bredow@thch.uni-bonn.de
* Corresponding author

Potential curves for adsorption of benzene on copper and gold surfaces

This supporting information contains potential curves for the adsorption of benzene on the Cu(111), Cu(100), Cu(110), Au(111), Au(100), and Au(110) surfaces. The data are obtained with the PBE-D3, PBE-D3(BJ), PBE-D3(ABC), RPBE-D3, RevPBE-D3, RevPBE-D3(BJ) methods.

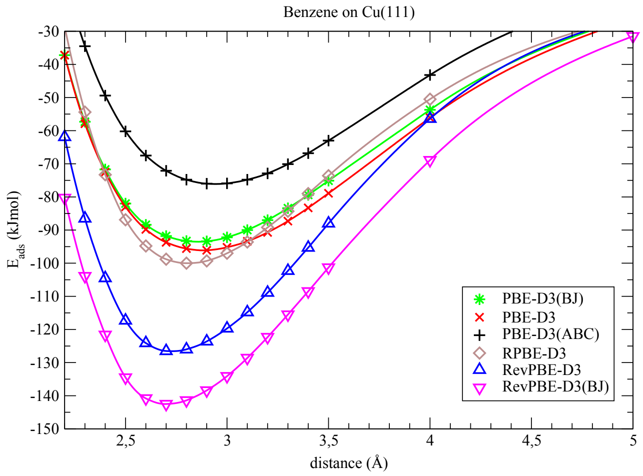

Figure 1: Potential curves for the adsorption of benzene on the Cu(111) surface.

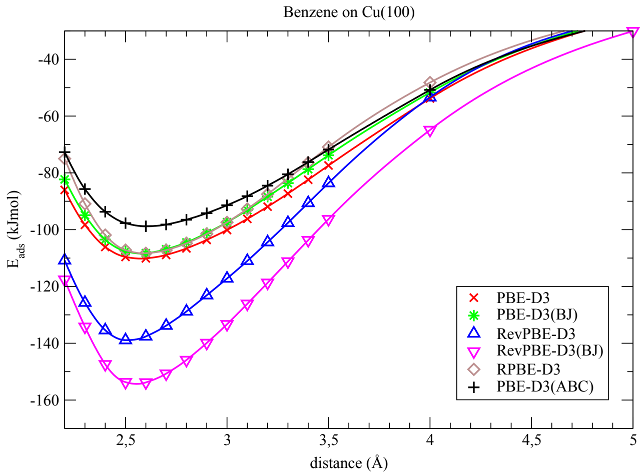

Figure 2: Potential curves for the adsorption of benzene on the Cu(100) surface.

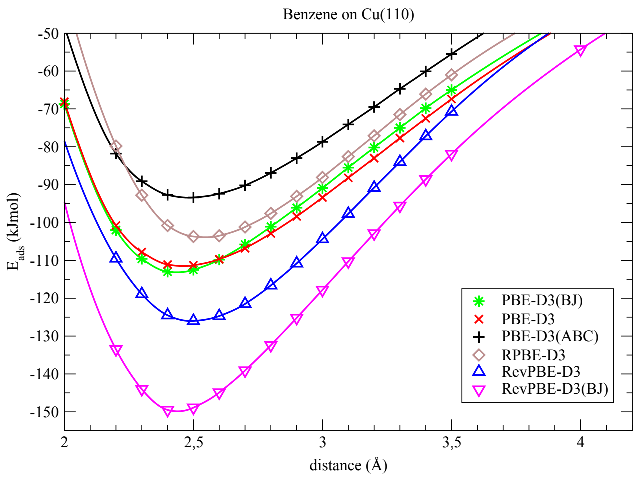

Figure 3: Potential curves for the adsorption of benzene on the Cu(110) surface.

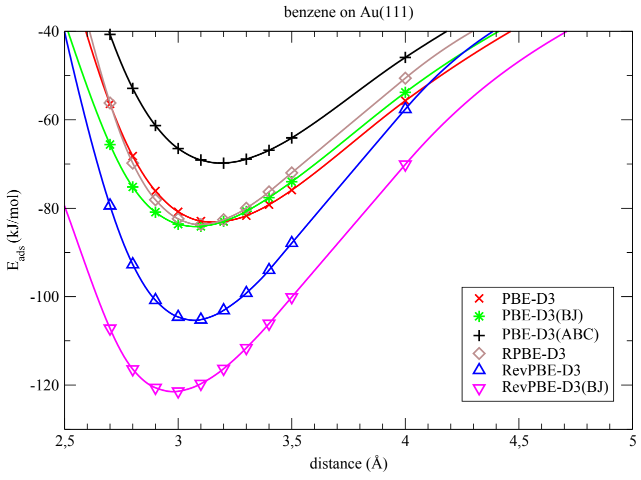

Figure 4: Potential curves for the adsorption of benzene on the Au(111) surface.

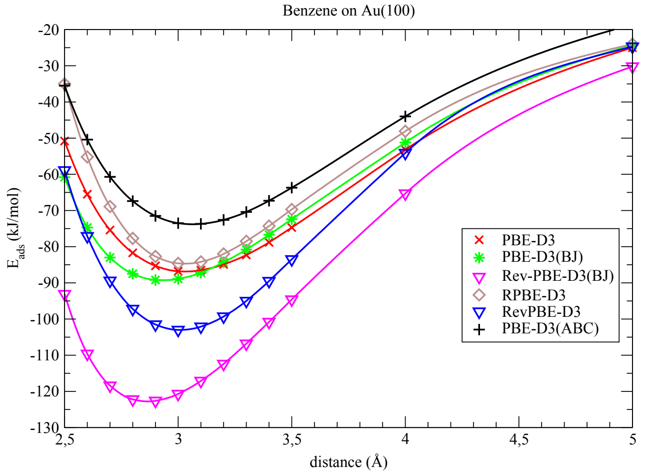

Figure 5: Potential curves for the adsorption of benzene on the Au(100) surface.

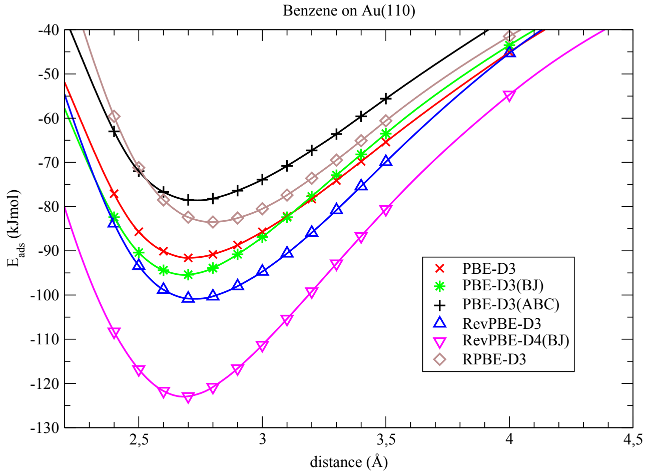

Figure 6: Potential curves for the adsorption of benzene on the Au(110) surface.
